# Eating Behaviors and Skin Carotenoids in Pregnant Women: The Moderating Influence of Depressive Symptoms and Income

**DOI:** 10.3390/nu17040739

**Published:** 2025-02-19

**Authors:** Lenka H. Shriver, Jessica M. Dollar, Mali Hosseinzadeh, Cheryl Buehler, Laurie Wideman, Esther M. Leerkes

**Affiliations:** 1 Department of Nutrition, University of North Carolina Greensboro, P.O. Box 26170, Greensboro, NC 27402-6170, USA; mphossei@uncg.edu; 2 Psychology & Kinesiology, Center for Women’s Health and Wellness, University of North Carolina Greensboro, P.O. Box 26170, Greensboro, NC 27402-6170, USA; jmdollar@uncg.edu; 3 Human Development and Family Studies, University of North Carolina Greensboro, P.O. Box 26170, Greensboro, NC 27402-6170, USA; cabuehle@uncg.edu (C.B.); emleerke@uncg.edu (E.M.L.); 4 Department of Kinesiology, University of North Carolina Greensboro, P.O. Box 26170, Greensboro, NC 27402-6170, USA; l_widema@uncg.edu

**Keywords:** eating behaviors, depression, fruit and vegetables, pregnancy, skin carotenoids, Veggie Meter

## Abstract

**Background**: Fruit and vegetable (FV) intake is critical for optimizing pregnancy outcomes. Several socio-demographic factors are associated with FV intake, but less is known about behavioral and mental health correlates. Furthermore, existing knowledge is largely based on self-reported FV intake. The current cross-sectional study examined depressive symptoms and income as moderators of the association between eating behaviors and skin carotenoids (FV status biomarker) in pregnancy. **Methods**: Participants living in an urban area of the south-astern part of the U.S. (N = 299) and who were in their third trimester of pregnancy were recruited for lab visits between 2019 and 2022 and completed the Dutch Eating Behavior Questionnaire and the Center for Epidemiological Studies Depression Scale. FV status was assessed using a pressure-mediated reflection spectroscopy to determine skin carotenoids. Hypotheses were tested via multiple regression. **Results**: There was an interaction between dietary restraint and depressive symptomatology such that greater restraint predicted higher skin carotenoids at low levels of depressive but not high levels. There was an interaction between restrained eating and family income in predicting skin carotenoids that was significant at high- but not low-income level. External and emotional eating did not predict skin carotenoids. **Conclusions**: Restrained eating might positively influence skin carotenoids during pregnancy. However, those who suffer from higher levels of depressive symptoms and/or live in lower-income households face additional barriers that might impede FV status. Further research is warranted to advance our understanding of the interplay between mental health, restrained eating and income on FV status in pregnancy.

## 1. Introduction

Fruit and vegetable (FV) consumption is associated with many health benefits, including prevention of cardiovascular disease and particular cancers, as well as reduced adiposity [1,2]. Thus, FV consumption is considered the cornerstone of a healthy diet, including during pregnancy [1,3]. Higher FV intake is associated with a lower risk of developing adverse pregnancy outcomes, such as excessive weight gain, maternal hyperglycemia, and a preterm delivery [4,5,6,7,8]. Furthermore, a systematic review of dietary intake among pregnant women found that high-fiber diets, which typically include adequate FV intake, are associated with a significantly lower risk of developing gestational diabetes during pregnancy [6]. However, there is little knowledge about pregnant women’s FV consumption and its correlates in this unique population and the existing findings are almost solely based on self-reported measures of FV intake (i.e., food frequencies; dietary recalls, screeners), which are subject to several types of errors and/or biases, such as social desirability and errors related to estimating portion sizes [9,10,11]. Although self-reported assessment of FV intake remains to be a common methodology in nutrition investigations due to its low cost and high feasibility, research utilizing innovative and advanced techniques, such as an assessment of skin carotenoids among pregnant women that reflect FV intake status over the past 2–3 months, is much needed in the current literature to address the aforementioned weaknesses [10].

Previous research has established several correlates of FV intake. For example, socio-demographic factors, such as race/ethnicity, low income, and living in areas with little access to healthy and affordable foods have been linked to lower FV intake [12,13,14]. However, most of the established barriers are difficult intervention targets given they are often embedded within the larger social and physical food environment [15]. More importantly, many individuals, including pregnant women, might still fail to meet the FV recommendations even when their income is higher and/or FV availability and accessibility are adequate [12]. Currently, there is a lack of studies examining modifiable correlates of FV consumption during pregnancy, especially those that would consider multiple levels of influence and could be used to better inform future effective interventions for pregnant women.

Eating behaviors (i.e., restrained eating, emotional eating, and external eating), might represent potential modifiable correlates of FV because they characterize individuals’ broader eating style and influence individual’s daily food choices [16]. Extreme restriction of food intake is harmful as it takes on many different forms, including dieting, fasting, and other types of unhealthy practices that individuals use for the purpose of weight loss. In contrast, restrained eating, as examined and measured by the Dutch Eating Behavior Questionnaire in the present study [16], has been defined as “successful” dietary restraint without subsequent bouts of overeating that individuals use to manage their diet [17]. Several studies found this type of restrained eating to be linked to a reduced level of excessive intake of kilocalories and less frequent snacking on unhealthy foods [18,19,20,21]. In our previous work, pregnant women with a higher level of moderate restrained eating were found to consume less added sugars and lower energy from dietary fat compared to pregnant women who engaged in less restraint [22]. 

Emotional and external eating are defined as eating without the presence of hunger, in response to emotional triggers (e.g., most commonly sadness or anxiety in emotional eating) and eating in response to external food cues (i.e., sight, smell, or taste in external eating) [23,24]. Previous research has found relatively consistent associations between emotional and external eating and negative diet-related outcomes. Specifically, individuals reporting higher levels of these eating behaviors were found to have a greater intake of palatable foods, such as high-fat and/or high-sugar snacks and beverages [19,25,26,27,28,29,30]. While previous studies have shown links between eating behaviors and negative dietary outcomes, such as a higher consumption of unhealthy foods and/or poor intake of some nutrients, little is currently known about the potential associations between emotional and external eating and skin carotenoids during pregnancy.

The role of depressive symptoms in establishing and maintaining a healthy diet has been increasingly recognized [31,32]. Recent studies show that higher levels of depressive symptoms, not only clinically diagnosed depression, are strongly associated with FV intake in adults and adolescents [33,34]. A recent systematic review of 61 studies found that a higher level of depressive symptoms was significantly correlated with a lower FV consumption [34]. It has been recognized that women in the reproductive years, including pregnant women, represent a population at the greatest risk for psychiatric disorders [35], and many individuals develop a depressive or other mental illness and/or experience a mental health incident during the prenatal period [36]. To date, very little knowledge exists on the links between dietary intake and depressive symptoms among pregnant women. Given the importance of FV status for pregnancy outcomes and the relatively high prevalence of mental health issues among women of reproductive age, a better understanding of the mechanisms through which mental health might influence FV status is greatly needed.

Higher levels of depressive symptoms appear to be associated with specific eating behaviors, including those examined in the current study [37,38]. For instance, Ugurlu et al. (2020) reported that individuals with more depressive symptoms engaged in greater levels of emotional and external eating compared to those with lower depressive symptoms [38]. Because emotional eating is typically triggered by negative emotions and “emotional eaters” are usually drawn to highly palatable foods, it is possible that within the context of depressive symptoms, emotional eating is likely to result in displacement of FV in the daily diet of some individuals [37]. Similarly, individuals who are more prone to engage in external eating and have higher levels of depressive symptoms might not have adequate motivation and/or desire to consume nutrient-dense foods, especially after consuming foods/beverages that are high in fat and/or sugar. Thus, depressive symptoms might play an important role in influencing the links between eating behaviors and FV intake status.

To date, it is unclear what the potential mechanisms of the associations between eating behaviors (i.e., restrained eating, emotional, and external eating), depressive symptoms, and FV intake are. Furthermore, most of the existing literature in this area comes from studies that rely on self-reports of FV intake, with even fewer specifically examining FV intake during pregnancy [22]. The current study fills these gaps by examining these associations in detail and by utilizing an objective measure of FV intake status among pregnant women using the Veggie Meter^®^ (Longevity Link Corp, Salt Lake City, UT, USA), which is an objective measure of FV intake status over the past 2–3 months [39]. The Veggie Meter^®^ device assesses skin carotenoids using pressure-mediated reflection spectroscopy and has been validated against the gold standard of FV intake status assessment, serum carotenoids, in previous research [40].

Thus, the main objective of the current cross-sectional study was to examine the role of depressive symptoms and family income in moderating the association between eating behaviors and skin carotenoids in a sample of racially/ethnically and socio-economically diverse pregnant women. Based on previous literature, we hypothesized that emotional and external eating is inversely associated, and restrained eating is positively correlated with FV intake during pregnancy. We also hypothesized that depressive symptoms and income moderate the association between eating behaviors and FV intake.

## 2. Materials and Methods

### 2.1. Study Design, Participants, and Procedures

Data for this cross-sectional investigation were collected as part of a large multidisciplinary longitudinal prospective study, titled the iGrow study that begun in 2019. The project followed pregnant women and their infants from the third trimester of pregnancy until the child reached the age of two, with participants residing in an urban area of the south-eastern part of the U.S. Details about the methodology and procedures for the longitudinal study are found elsewhere [41]. Since skin carotenoid assessment was not part of the original funded study protocol and was added later, detailed procedures relate to the use of the Veggie Meter^®^ device in the current cross-sectional investigation are described below. The inclusion criteria for the overall iGrow study were as follows: (1) maternal age of 18 years or older; (2) expecting a singleton birth; (3) being fluent in oral and written English; and (4) planning to remain in the region for at least 3 years. Originally, 542 potential participants expressed interest and verbally agreed to participate in the longitudinal study. From those, 299 individuals followed through and provided at least some data during the project duration. The sample size for the current study was based on convenience. However, the power calculation for the overall iGrow study indicated that a sample size of 250 would distinguish between a good fitting model and an adequately fitting model (RMSEA = 0.065) at a power level of 0.80. Because the power calculations used for the overall study were based on significantly more complex and longitudinal aims than the moderation analyses presented in the current cross-sectional study, the sample size of 299 was deemed adequate to meet the objectives of the analyses presented here.

The participants included in the current study were biological females and, thus, the terms “pregnant women” and “maternal” are used from here on out. All eligible participants who provided at least some data during the prenatal data collection wave during the last few weeks of their pregnancy were included in the analyses presented here. The data were collected during a prenatal laboratory visit and via questionnaires completed via Qualtrics prior to the prenatal visit. All prenatal visits were completed between 2019 and 2022. During the laboratory visit, participants were asked to complete anthropometric measurements (e.g., height, weight) and completed skin carotenoid measurement. Informed consent was obtained from each participant prior to data collection. This research was conducted in accordance with the ethical principles for research with human participants. All participants provided written informed consent prior to any data collection. The study was reviewed and approved by the Institutional Review Board of the University of North Carolina at Greensboro prior to recruitment and data collection (date of approval: 16 April 2018; protocol #: 18-0198).

### 2.2. Sociodemographic Characteristics

Participants were asked to complete questionnaires about their age, race/ethnicity, household characteristics, income, education, and other variables. Income-to-needs ratio was calculated by dividing the total annual household income by its corresponding poverty threshold based on the number of household members and the year of the reported income. We used the 2018 and 2019 threshold values that were published by the U.S. Census Reports in the Poverty Thresholds [42].

### 2.3. Anthropometrics

Self-reported weight prior to pregnancy and height measured during the prenatal lab visit were used to calculate pre-pregnancy Body Mass Index (BMI) (as calculated by weight in kilograms/height in m^2^). For descriptive purposes, participants were also categorized into weight status categories according to their pre-pregnancy BMI: (1) underweight (BMI < 18.5 kg/m^2^); (2) healthy weight (BMI = 18.5–24.9 kg/m^2^); (3) overweight (BMI = 25.0–29.9 kg/m^2^); and (4) obese (BMI ≥ 30.0 kg/m^2^) based on the guidelines developed by the Centers for Disease Control and Prevention [43]. Additional information related to established correlates of skin carotenoids, including cigarette use and vitamin/mineral supplement use during pregnancy and other information, was collected via self-reported online questionnaires around the time of the scheduled lab visit.

### 2.4. Skin Carotenoids

Skin carotenoid levels, a proxy of FV intake status, were assessed using the Veggie Meter^®^ device. The device utilizes a pressure-mediated reflection spectroscopy to capture skin Veggie Meter^®^ carotenoids via a brief and non-invasive finger scan [39,44]. The device is also programmed with an algorithm to correct for pigmentation from melanin and has been validated in racially/ethnically diverse populations [40]. Skin carotenoid levels were measured in triplicates and the mean of the 3 measurements was used in the final analyses. The possible range of skin carotenoid levels is 0 to 800, with a higher score indicating a higher FV intake over the past 3 months, thus capturing the FV intake of the participants during the latter part of their pregnancy.

### 2.5. Eating Behaviors

The 33-item Dutch Eating Behavior Questionnaire (DEBQ) was used to assess dietary restraint, emotional eating and external eating. The response format ranged from “never = 1” to “very often = 5” [16]. This measure was previously validated and widely utilized in research, including studies with pregnant women [45,46,47]. Participants were asked to provide responses to 10 restraint eating items (α = 0.92), 13 emotional eating items (α = 0.96), and 10 external eating items (α = 0.83). A higher score indicated a greater degree of engaging in the specific behavior. Original wording for one item related to “eating less in response to weight gain” in the restraint eating subscale was modified to “before you were pregnant” for the purposes of the study because weight gain is expected and desired during pregnancy.

### 2.6. Depressive Symptoms

Participants were asked to complete the 20-item Center for Epidemiological Studies Depression Scale (CES-D) [48]. Participants responded by indicating how they felt during the previous week on a 4-point scale ranging from 0 = rarely/never to 3 = most of the time. The measure has been validated and widely used in previous research [49]. After appropriate items were reversed coded, the responses were summed (α = 0.89) and the depressive symptom score was used as a continuous variable. For descriptive purposes, participants were also classified as having clinically elevated depression, as a score of 16 or higher is the clinical cut off (16% in this sample) [48].

### 2.7. Statistical Analyses

Preliminary analyses were conducted in SPSS (Version 29.0, IBM, Chicago, IL, USA) and descriptive statistics and potential covariates are displayed in Table 1. Primary analyses were conducted using Mplus, version 8.11 [50]. Full information maximum likelihood (FIML) was used to handle missing data, which minimizes estimation bias [51]. The covariates of maternal age, education, income-to-needs ratio, race/ethnicity, smoking during pregnancy, season when skin carotenoid level was assessed, and use of multivitamin supplement were selected based on theoretical and empirical evidence that each might influence skin carotenoids [40]. A multiple regression analysis was used to examine the moderating effects of depressive symptoms and family income on the associations between all three eating behaviors (and dietary restraint, emotional eating, and external eating) and skin carotenoid level (as measured by Veggie Meter^®^), adjusted for covariates. We used a bias-corrected bootstrapping procedure (10,000 draws). Variables were grand-mean-centered, and an interaction term was created to be used in analyses. Interactions that were significant or approached significance at *p* < 0.10 [52] were probed by calculating simple slopes at one standard deviation above and below the mean level of the moderator [53].

## 3. Results

Data from 299 pregnant women who provided at least some data during the prenatal visit were included in the analyses. The sample characteristics are presented in Table 1. Participants ranged in age from 18 to 49 years. Using the pre-pregnancy BMI, participants were categorized based on their weight status as follows: underweight (n = 8; 2.7%); healthy weight (n = 109; 36.5%); overweight (n = 84; 28.1%); and obese (n = 97; 32.4%). The sample was racially/ethnically and socio-economically diverse: 29% identified as Non-Hispanic Black and an additional 19% identified as either Hispanic, multi-racial, or other [Table 1]. Approximately 16% of the participants reported clinical levels of depressive symptoms. The mean values of eating behaviors as measured by DEBQ in the sample (possible score range 1–5) were as follows: dietary restraint (2.26 ± 0.78); emotional eating (2.19 ± 0.92); and external eating (2.93 ± 0.61). Missing data on key variables were minimal (skin carotenoids, n = 17; eating behaviors, n = 10; depressive symptoms, n = 20).

Bivariate correlations revealed that skin carotenoids were positively associated with age, income, education, and dietary restraint and negatively associated with pre-pregnancy BMI, cigarette use, and depressive symptoms [Table 2]. Analysis of variance (ANOVA) showed no significant differences in skin carotenoid level by race/ethnicity (*p* = 0.26), multivitamin supplement use during pregnancy (*p* = 0.16), or season of the year (*p* = 0.72).

**Figure 1:**
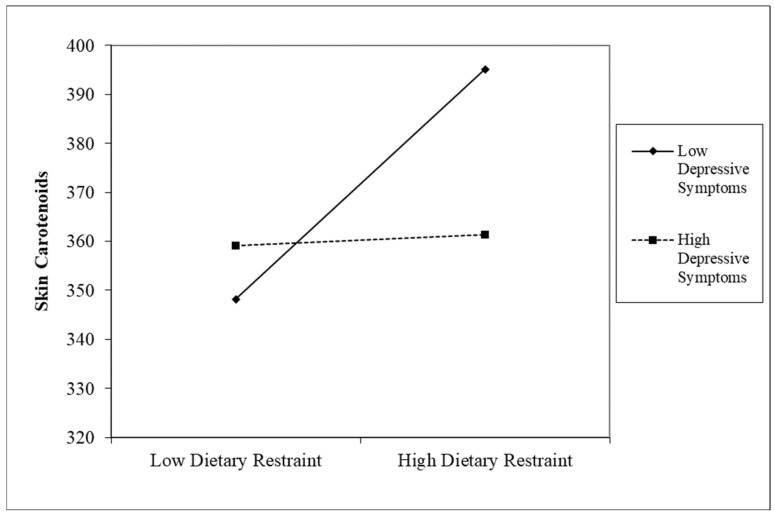
The association between maternal dietary restraint and skin carotenoids at low and high levels of depressive symptoms.

After controlling for age, smoking, season of the year, pre-pregnancy BMI, race/ethnicity, education, and vitamin/mineral supplement use, significant main effects for dietary restraint (*β* = 0.17, *p* < 0.01) and family income (*β* = 0.17, *p* < 0.01) emerged. However, these main effects were subsumed under significant interaction effects. First, a depressive symptomatology significantly moderated the association between dietary restraint and skin carotenoid levels (*β* = −0.11, *p* < 0.05, 95%*CI_lower_* = −0.22, 95%*CI_upper_* = −0.01; Figure 1).

**Figure 2:**
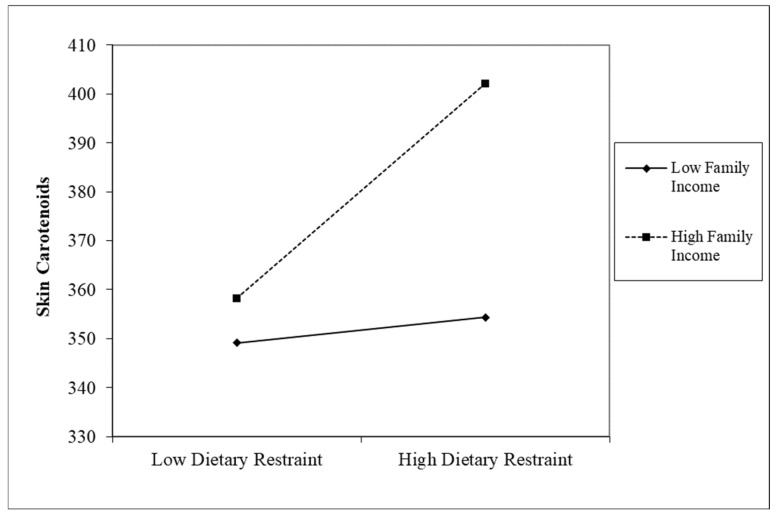
The association between maternal dietary restraint and skin carotenoids at low and high levels of family income.

In the next step, we followed procedures for probing interactions as outlined by Aiken et al. [53]. First, the continuous variables of interest (i.e., eating behaviors, depressive symptoms, income) were grand-mean centered before interaction terms were created. Second, the interactions that were significant at *p* < 0.05, or approached significance at *p* < 0.10 [52], were probed by calculating simple slopes at one standard deviation above and below the mean level of the moderator (i.e., depressive symptoms and income in the analyses presented here) [53]. Using this approach, the follow up analyses indicated that that this association was significant at low levels of depression (*β* = 0.29, *p* < 0.001), but not high levels of depression (*β* = 0.06, *p* > 0.10). For women with low levels of depressive symptomatology, their skin carotenoid levels increased as dietary restraint increased. In addition, there was a significant interaction between dietary restraint and family income (*β* = 0.12, *p* = 0.07, 90%*CI_lower_* = 0.01, 90%*CI_upper_* = 0.24; Figure 2) in predicting skin carotenoid levels. This relation was significant at high levels of income (*β* = 0.28, *p* < 0.001), but not low levels of income (*β* = 0.08, *p* > 0.10). For women with a high family income, their skin carotenoid levels increased as dietary restraint increased.

## 4. Discussion

The current study examined the associations between eating behaviors and skin carotenoids, a biomarker of FV intake among pregnant women. Although emotional and external eating were not associated with skin carotenoids, dietary restraint was found to be an important correlate of pregnant women’s FV intake status in our sample. The biggest contribution of the current study is that we identified ways in which associations between restrained eating and skin carotenoids change within the context of both depressive symptoms and family income. Thus, findings presented here might inform and help individualize future efforts aimed at improving FV consumption among pregnant women, with the end goal of reducing risks of adverse pregnancy-related outcomes associated with limited FV intake.

Our findings that greater restraint is associated with higher FV intake is consistent with some studies that linked “successful/moderate” dietary restraint to positive diet-related outcomes [20,22]. For instance, our recent study found that pregnant women who engaged in higher dietary restraint reported reduced intake of total added sugars and less energy from fat [22]. Given our findings, it is possible that pregnant women with a higher dietary restraint are successful in consuming FV in place of other less nutrient-dense foods, and thus might be able to maintain.

However, it is important to note that the positive influence of dietary restraint on skin carotenoids in our study was only found among pregnant women living in higher-income households. Given that low-income families typically face a variety of financial, environmental, and social barriers that negatively impact opportunities to consume more FV, it is likely that engaging in more dietary restraint might not be sufficient to increase FV in the light of all these daily barriers. Low socioeconomic status has been long recognized as an important barrier to FV consumption across the lifespan [15] and its negative impact is also evident from the results of the nationally representative 2019 Behavior Risk Factor Surveillance Survey (BRFSS) in which individuals living in poverty or close to the poverty level reported some of the lowest FV intake in the U.S. [13]. Therefore, pregnant women living in lower-income households are more likely to be food insecure, live in food deserts with less access to fresh FV, be less likely to have reliable and regular transportation for grocery shopping, and/or might have less time to prepare meals/snacks that include FV [54,55].

As shown in the current study, dietary restraint might serve as a useful tool for increasing FV among pregnant women, but only among pregnant women who have plenty of resources and live in middle–high-income households. At the same time, the urgent need to address major barriers to FV consumption for low-income populations remains despite increasing efforts to do so in recent years [56,57]. For example, a study by Trapl et al. [57] utilized a monthly produce prescription and provided nutrition education at prenatal visits to low-income pregnant women. Findings from this mixed-method intervention study showed that participants not only took advantage of the produce prescriptions, but the perceived value of the program was high, and they valued the opportunity to receive nutrition information and talk about their diet and shopping habits. Thus, further innovative interventions that address some of the issues surrounding access and availability of FV to disadvantaged populations, including those who are pregnant, remain warranted.

In our study, the influence of restrained eating on skin carotenoids was pronounced among women who reported low levels of depressive symptomatology. Previous evidence shows that depression is associated with lowered energy and lower motivation to make healthy lifestyle choices. Thus, it is likely that women experiencing mental health issues, especially in addition to the increasing changes and potential stressors associated with pregnancy, might find it more difficult to engage in behaviors that promote health, such as higher FV intake and/or planning healthy snacks/meals. Further, there is growing evidence of trends for increased mental illness, including among pregnant women [58], which highlights the significance of our findings that mental health influences FV intake in pregnant women and thus, might directly impact pregnancy-related health outcomes. On the other hand, our findings suggest that among pregnant women with low levels of depressive symptomatology, increases in restrained eating are important in predicting observed skin carotenoid levels. Our finding adds to the body of literature suggesting that “successful” restrained eating, not unhealthy and extreme types of restriction, is linked with reduced levels of excessive intake of kilocalories, less frequent snacking on unhealthy foods [18,19,20,21] and lower self-reported consumption of added sugars and energy from fat among pregnant women [22].

Contrary to our expectations, neither emotional nor external eating were associated with skin carotenoid levels in our sample of pregnant women. This finding was surprising especially regarding emotional eating since emotional eaters tend to report higher intake of low nutrient dense foods (i.e., junk foods) that might displace more nutrient-dense foods such as FV in their diet, such as FV [59]. However, overconsumption of energy, fat and/or sugar due to high intake of palatable foods (i.e., junk food) might also occur while FV consumption is similar to the intake of individuals who engage in similar levels of emotional and/or external eating. In other words, the impact of emotional and external eating might be largely observed in the excessive intake of unhealthy nutrients and/or excessive energy, but not necessarily in the reduced intake of healthy foods (i.e., FV), which also explains the positive associations found between emotional and external eating and obesity in previous research [59].

Most adults in the U.S. consume less FV than recommended by the current Dietary Guidelines for Americans [3], with only 12.3% meeting the fruit and 10% meeting the vegetable recommendations [13]. Similar trends have been observed in pregnant women, with a substantial proportion failing to meet the recommendations for nutrients primarily found in FV, suggesting poor FV consumption [9]. Some argue; however, that becoming pregnant might lead to positive changes in food choices and thus the greater intake of healthy foods [10,60]. However, these findings come from studies that utilized self-reported FV intake data and thus it is difficult to confirm to what degree improvements in FV intake occurred [11]. Thus, the present study represents an essential step towards clarifying these trends in order to better inform future intervention efforts. In our sample of pregnant women, the mean skin carotenoid score was 257, with nearly 2/3 of the sample having values under 280. Considering that each 100 units of the Veggie Meter^®^ score corresponds to approximately 1 cup of FV per day, our sample was well below the minimum recommendation of consuming at least three cups of FV per day [3,61]). Although this is the first study to assess skin carotenoids in a relatively large sample of pregnant women, we did expect the levels to be higher compared to most previously reported skin carotenoid levels from non-pregnant adult samples [56,61]. Our findings might be, at least partly, explained by the fact that our sample included a relatively large proportion of lower-income families and those living in an urban area with potentially limited access to fresh FV, compared to other samples with higher skin carotenoids with a higher education [62] and/or access to farmer’s markets on a regular basis [56].

Although not statistically significant, we found some interesting trends in FV intake status between the different racial/ethnic groups represented in our sample. While Hispanic White women appeared to consume the greatest amount of FV in the past 2–3 months which was the time period captured by the skin carotenoid assessment, the lowest skin carotenoid values were found among the Non-Hispanic Black women in our sample. These trends are consistent with previous studies that also used the Veggie Meter^®^ and reported relatively low levels of skin carotenoids in samples of predominantly African American adult participants [63,64]. These trends could be, at least partly, explained by a variety of factors, including possible racial/ethnic and cultural differences that have been shown to underline food-related patterns across different racial/ethnic and cultural groups in previous research [65,66]. While this was not the aim of the current study, it is important to further investigate the role of race/ethnicity and cultural food-related practices on FV intake among pregnant women.

The current study has several strengths that should be noted. First, the current study contributes to the literature on FV intake among pregnant women by utilizing an objective biomarker of FV consumption that has been validated against the gold standard of FV intake status (i.e., plasma carotenoids) [67]. Second, in addition to skin carotenoids being a more accurate measure of FV consumption, skin carotenoid levels also reflect FV consumption over the past 2–3 months, thus capturing habitual intake over a longer period of time compared to most other studies, especially among pregnant women where studies are limited [10]. Third, our analyses are based on data from a relatively large sample of pregnant women (N = 299) with diverse sociodemographic backgrounds, with nearly half self-identifying as non-Hispanic Black, Hispanic or other/multiracial. Fourth, findings from our study contribute to the growing number of studies that utilize the Veggie Meter^®^ as an objective measure of FV intake and thus a large effort to create a data repository on skin carotenoid levels across different populations, including pregnant women.

There are also several limitations that should be noted. First, our sample, although relatively large, does not represent FV intake status of pregnant women across the U.S. Because skin carotenoids represent an objective measure of FV intake status over 2–3-month period, assessment of this biomarker in a nationally representative sample of pregnant women in the U.S. would be very useful for assessing FV intake more accurately and also comparing pregnant women’s FV consumption across studies. Second, data presented here came from a larger longitudinal study that recruited and scheduled prenatal visit with participants over a 2-year period, including during the COVID-19 pandemic. Thus, the skin carotenoid assessment was conducted across different seasons of the year. Although we controlled for the effect of season in the primary analyses, the skin carotenoids detected in our sample might have been influenced by the timing of the visits, and also by broader circumstances of the global pandemic.

## 5. Conclusions

Diets rich in FV help optimize pregnancy-related outcomes for both the mother and the infant. Our finding that higher restrained eating is associated with better FV intake status is important, especially since hormonal changes during pregnancy often increase women’s cravings for high-sugar and/or fat foods that might misplace FV in their prenatal diet. However, our findings also highlight that consuming more FV, perhaps by being aware of nutrient content and/or replacing high-energy dense foods with FV, is much more difficult for pregnant women who have additional barriers, such as experiencing a higher level of depressive symptoms and/or living in a low-income household. Although many new efforts have recently focused on improving availability and accessibility of FV to low-income families, much less attention has been given to the role of eating behaviors and mental health on establishing and/or maintaining a healthy diet during pregnancy. Thus, prenatal care should include screening of pregnant women for depressive symptoms, diet quality, as well as eating behavior patterns. Such a holistic approach to prenatal care would allow for identifying the target foci for potential interventions and thus provide women with more individualized support that could optimize their FV consumption during pregnancy.

## Figures and Tables

**Table 1:** Study Descriptives (n = 299).

Characteristics/Variables	N	M (SD)/%
Age, years	299	29.73 (5.55)
Pre-pregnancy BMI ^1^	299	28.52 (7.30)
Income-to-needs ratio ^2^	290	3.50 (2.96)
Cigarette Use ^3^	297	1.08 (0.40)
Vitamin/Mineral Use	299	
Yes		163 (54.5)
No		136 (45.5)
Race/Ethnicity	299	
Non-Hispanic White		157 (52.5)
Non-Hispanic Black		86 (28.8)
Hispanic/Other/Multiracial		56 (18.7)
Educational attainment	295	
≤High school diploma/GED		47 (15.9)
Some college		57 (19.3)
2-year/4-year college degree		98 (33.2)
Post graduate work/degree		93 (31.5)
Depressive symptoms ^4^	279	12.13 (78.5)
Skin Carotenoid Score ^5^	282	257.72 (91.05)

^1^ Calculated as pre-pregnancy weight in kg/height in m^2^; ^2^ determined by dividing total annual household income by its corresponding poverty threshold based on the number of household members and the year of the income that was reported (2018–2019) [42]; ^3^ mean number of cigarettes used during pregnancy; ^4^ mean value of depressive symptoms assessed via the 20-item Center for Epidemiological Studies Depression Scale; ^5^ skin carotenoids measured by Veggie Meter^®^ in triplicates, possible score 0–800).

**Table 2:** Bivariate Correlations among Study Variables.

Variable	1	2	3	4	5	6	7	8	9	10
1. Skin carotenoids	-									
2. Age, years	0.12 *	-								
3. Pre-pregnancy BMI	−0.37 **	0.04	-							
4. Income-to-needs ratio	0.30 **	0.29 **	−0.23 **	-						
5. Education	0.17 **	0.49 **	−0.12 *	0.59 **	-					
6. Cigarette use	−0.15 **	−0.02	0.00	−0.19 **	−0.21 **	-				
7. Depressive symptoms	−0.17 **	−0.20 **	0.16 **	−0.28 **	−0.27 **	0.23 **	-			
8. Dietary restraint	0.17 **	0.22 *	0.07	0.26 **	0.32 **	−0.16 **	0.06	-		
9. Emotional eating	−0.06	0.07	0.14 *	0.16 *	0.23 **	−0.08	0.24 **	0.29 **	-	
10. External eating	0.03	0.02	−0.02	0.14 *	0.14 *	−0.07	0.15 **	0.23 **	0.64 *	-

* *p* < 0.05, ** *p* < 0.01.

## Data Availability

The data presented in this study are available on request from the corresponding author (LHS) due to privacy reasons.
